# Supplementary material for: Host glutathione is required for Rickettsia parkeri cell division and intracellular survival
Source: Nat Commun. 2025 Jul 1;16:5547. doi: 10.1038/s41467-025-60509-7 (PMC12216324; doi:10.1038/s41467-025-60509-7)
Supplement: Supplementary file 1 — Supplementary Information [file 41467_2025_60509_MOESM1_ESM.pdf]

Host glutathione is required for *Rickettsia parkeri* cell division and intracellular survival.

Supplementary Figures:

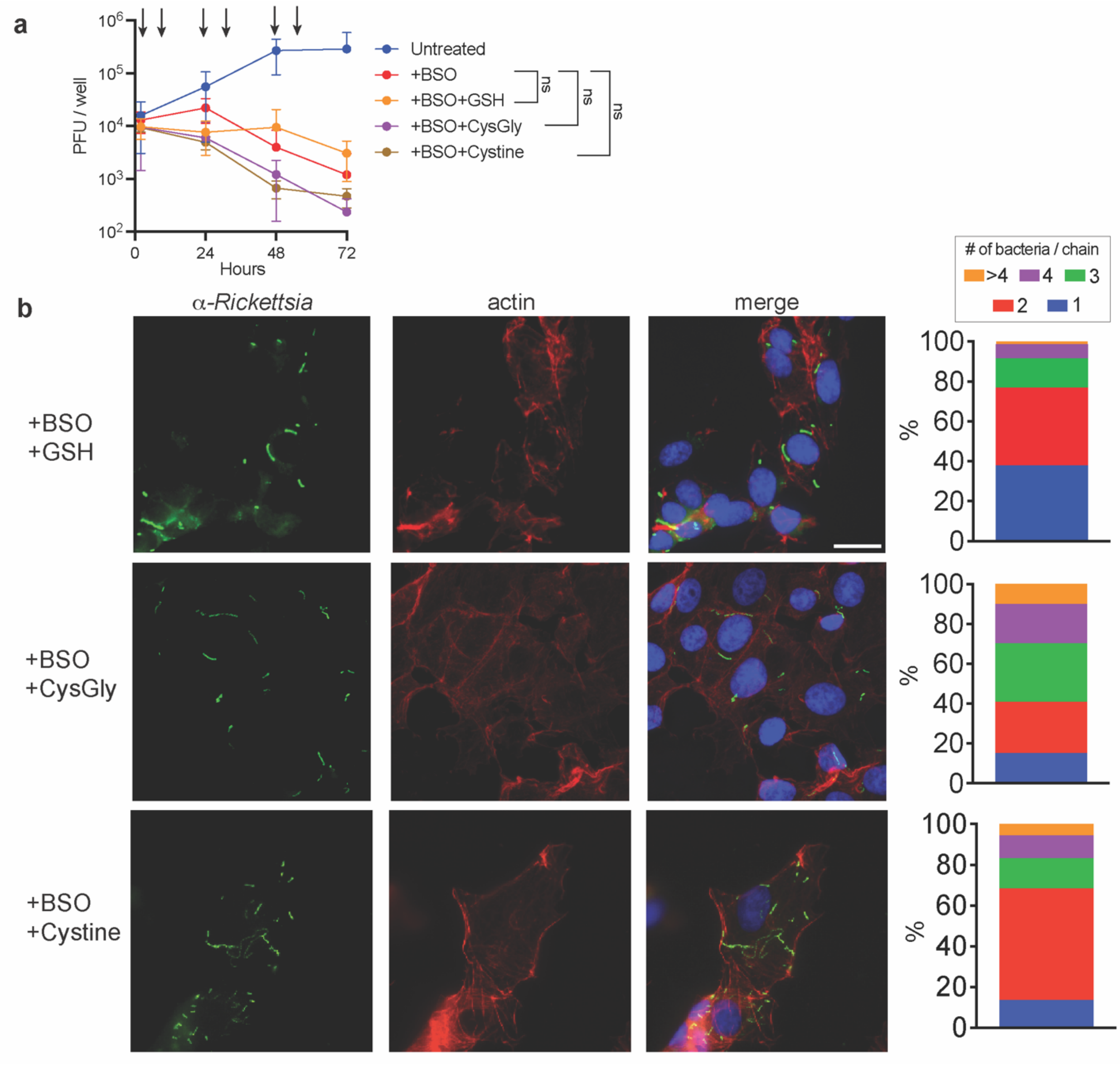

Supplementary Figure 1: GSH, CysGly and cystine do not rescue survival or chaining defects of *R. parkeri* in BSO-treated cells

a) PFUs of *R. parkeri* in BMDMs in the presence of the indicated metabolites. Data are combined from six separate experiments and are means $\pm$ SD. Statistics are two-way T-tests at 72 hpi, ns=not significant. **b**) Representative images and quantification (right) of *R. parkeri* in Vero cells at 48 hpi in the presence of the indicated metabolites, 2 mM GSH, 50 µM CysGly, 5 mM cystine, added 1 hpi. Scale bar = 20 µm. Green = α-*Rickettsia* antibody; red = phalloidin (actin), blue = DAPI. 3 separate experiments were counted, using 3 images per experiment and >200 bacteria total. BSO (2 mM) was added overnight prior to infection.

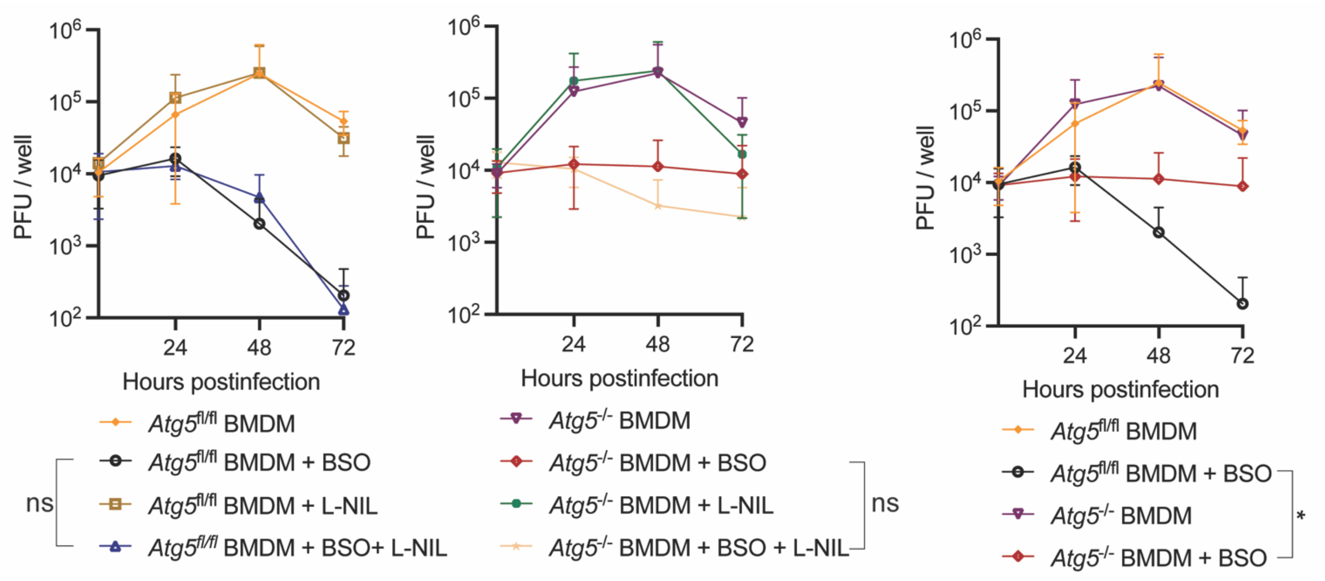

Supplementary Figure 2: Inhibiting iNOS does not rescue *R. parkeri* survival upon BSO treatment in the absence or presence of ATG5.

The indicated BMDMs were infected at an MOI of 1 and PFUs were monitored over time. BSO was added overnight prior to infection at 2 mM. L-NIL was added 1 hpi at 1 mM. Data are the compilation of six separate experiments and are expressed as means $\pm$ SD. Statistics used a two-way T test at 72 hpi, *p<.05, ns=not significant.

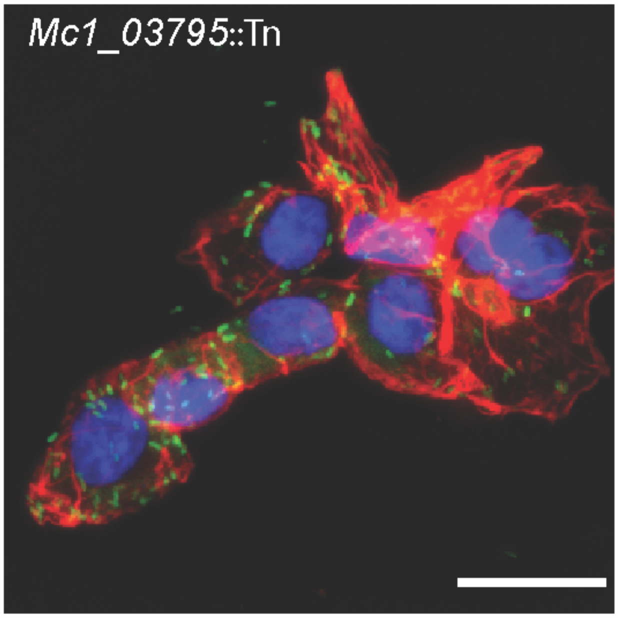

Supplementary Figure 3: A transposon insertion mutant in the predicted glutathione transferase A (GstA) has normal morphology and actin-based motility.
Representative image of the *gstA*::Tn *R. parkeri* mutant in Vero cells at 48 hpi, at 60x, scale bar = 20 μm.

Figure	Comparison	P value
1a	Veros vs. Veros + BSO	****p<0.0001
1c	Untreated vs. + BSO	p=0.6281
1i	All Comparisons	****p<0.0001
1j	Untreated vs. Treated	****p<0.0001
2d	Untreated vs. +BSO to Veros	**p=0.0068
3a	Veros vs. Veros + BSO	****p<0.0001
	BMDMs vs. BMDMs + BSO	***p=0.0006
	Veros + BSO vs. BMDMs + BSO	**p=0.0073
3c	Untreated vs. + BSO	p=0.3039
3d	WT untreated vs. WT + BSO	p=0.9689
	Casp 1/11$^{-/-}$ untreated vs. *Casp 1/11*$^{-/-}$ + BSO	***p=0.0006
3e	Uninfected vs. Uninfected + BSO	p=0.9978
	Infected vs. Infected + BSO	p=0.8458
	Uninfected vs. Infected	****p<0.0001
4b	Vehicle vs. +BSO	*p=0.0457
4c	*Atg5*$^{fl/fl}$ BMDMs + BSO vs. *Atg5*$^{-/-}$ BMDMs + BSO 48hpi	*p=0.0333
	Atg5$^{fl/fl}$ BMDMs + BSO vs. *Atg5*$^{-/-}$ BMDMs + BSO 72hpi	*p=0.0183
4d	+ BSO vs. + BSO + 3MA 24hpi	*p=0.0299
	+ BSO vs. + BSO + 3MA 48hpi	**p=0.0056
	+ BSO vs. + BSO + 3MA 72hpi	*p=0.010
5a	All comparisons	****p<0.0001
5c	Positive control vs. untreated in A549s	*p=0.0105
	Untreated vs. + BSO in A549s	p=0.8398
	Untreated vs. + BSO in Veros	p=0.6816
	All other comparisons	****p<0.0001
5d	+BSO vs. +H_2O_2	****p<0.0001
5f	Positive Control vs. Untreated	**p=0.0011
	Infected vs. Infected + BSO	*p=0.0361
	Uninfected vs. Uninfected + BSO	p=0.2982
	Uninfected vs. Infected	p=0.0579
5g	WT BMDM vs. WT BMDM + BSO	***p=0.0007
	WT BMDM + BSO vs. WT BMDM + BSO + L-NIL	p=0.1603
S1a	+BSO vs. +BSO + GSH	p=0.1381
	+BSO vs. +BSO + CysGly	p=0.7129
	+BSO vs. +BSO + Cystine	p=0.4554
S2	*Atg5*$^{fl/fl}$ BMDMs + BSO vs. *Atg5*$^{fl/fl}$ BMDMs + BSO + L-NIL	p=0.5716
	Atg5$^{-/-}$ BMDMs + BSO vs. *Atg5*$^{-/-}$ BMDMs + BSO + L-NIL	p=0.2576

	Atg5$^{fl/fl}$ BMDMs + BSO vs. *Atg5*$^{-/-}$ BMDMs + BSO 72hpi	*p=0.0183

Supplementary Table 1: p value for all figures.
