# Supplementary material for: Host glutathione is required for Rickettsia parkeri cell division and intracellular survival
Source: Nat Commun. 2025 Jul 1;16:5547. doi: 10.1038/s41467-025-60509-7 (PMC12216324; doi:10.1038/s41467-025-60509-7)
Supplement: Supplementary file 2 — Reporting Summary [file 41467_2025_60509_MOESM2_ESM.pdf]

nature portfolio

Corresponding author(s): Thomas P Burke

Last updated by author(s): 2025-22-05

Reporting Summary

Nature Portfolio wishes to improve the reproducibility of the work that we publish. This form provides structure for consistency and transparency in reporting. For further information on Nature Portfolio policies, see our Editorial Policies and the Editorial Policy Checklist.

Statistics

For all statistical analyses, confirm that the following items are present in the figure legend, table legend, main text, or Methods section.

n/a	Confirmed	
☐	☒	The exact sample size (*n*) for each experimental group/condition, given as a discrete number and unit of measurement
☐	☒	A statement on whether measurements were taken from distinct samples or whether the same sample was measured repeatedly
☐	☒	The statistical test(s) used AND whether they are one- or two-sided<br>*Only common tests should be described solely by name; describe more complex techniques in the Methods section.*
☒	☐	A description of all covariates tested
☐	☒	A description of any assumptions or corrections, such as tests of normality and adjustment for multiple comparisons
☐	☒	A full description of the statistical parameters including central tendency (e.g. means) or other basic estimates (e.g. regression coefficient) AND variation (e.g. standard deviation) or associated estimates of uncertainty (e.g. confidence intervals)
☐	☒	For null hypothesis testing, the test statistic (e.g. *F*, *t*, *r*) with confidence intervals, effect sizes, degrees of freedom and *P* value noted<br>*Give P values as exact values whenever suitable.*
☒	☐	For Bayesian analysis, information on the choice of priors and Markov chain Monte Carlo settings
☒	☐	For hierarchical and complex designs, identification of the appropriate level for tests and full reporting of outcomes
☒	☐	Estimates of effect sizes (e.g. Cohen's *d*, Pearson's *r*), indicating how they were calculated

Our web collection on statistics for biologists contains articles on many of the points above.

Software and code

Policy information about availability of computer code

Data collection	Data were collected and stored in Excel or Prism. No custom code was used.
Data analysis	Data including statistics were analyzed in Graphpad Prism. No custom code was used

For manuscripts utilizing custom algorithms or software that are central to the research but not yet described in published literature, software must be made available to editors and reviewers. We strongly encourage code deposition in a community repository (e.g. GitHub). See the Nature Portfolio guidelines for submitting code & software for further information.

Data

Policy information about availability of data

All manuscripts must include a data availability statement. This statement should provide the following information, where applicable:

- Accession codes, unique identifiers, or web links for publicly available datasets
- A description of any restrictions on data availability
- For clinical datasets or third party data, please ensure that the statement adheres to our policy

The manuscript includes a data availability section. R. parkeri strains were authenticated by whole genome sequencing and are available in the NCBI Trace and Short-Read Archive; Sequence Read Archive (SRA), accession number SRX4401164. Source data are provided in the Dryad repository doi:10.5061/dryad.905qfttvz.

Research involving human participants, their data, or biological material

Policy information about studies with human participants or human data. See also policy information about sex, gender (identity/presentation), and sexual orientation and race, ethnicity and racism.

Reporting on sex and gender	na
Reporting on race, ethnicity, or other socially relevant groupings	na
Population characteristics	na
Recruitment	na
Ethics oversight	na

Note that full information on the approval of the study protocol must also be provided in the manuscript.

Field-specific reporting

Please select the one below that is the best fit for your research. If you are not sure, read the appropriate sections before making your selection.

☒ Life sciences ☐ Behavioural & social sciences ☐ Ecological, evolutionary & environmental sciences

For a reference copy of the document with all sections, see nature.com/documents/nr-reporting-summary-flat.pdf

Life sciences study design

All studies must disclose on these points even when the disclosure is negative.

Sample size	Each sample was the average of two or more technical replicates. At least 3 independent experiments were performed for each experiment, as described in each figure legend
Data exclusions	No data were excluded
Replication	At least 3 independent experiments were performed.
Randomization	Samples in tissue culture plates were randomly assigned
Blinding	Samples for image analysis were blinded

Reporting for specific materials, systems and methods

We require information from authors about some types of materials, experimental systems and methods used in many studies. Here, indicate whether each material, system or method listed is relevant to your study. If you are not sure if a list item applies to your research, read the appropriate section before selecting a response.

Materials & experimental systems

n/a	Involved in the study	
☐	☒	Antibodies
☐	☒	Eukaryotic cell lines
☒	☐	Palaeontology and archaeology
☐	☒	Animals and other organisms
☒	☐	Clinical data
☒	☐	Dual use research of concern
☒	☐	Plants

Methods

n/a	Involved in the study	
☒	☐	ChIP-seq
☒	☐	Flow cytometry
☒	☐	MRI-based neuroimaging

Antibodies

Antibodies used	The Antibody Supplier, Name, Catalog Number , Lot Number, and Dilution are below<br>R. parkeri was detected using mouse anti-Rickettsia 14-13 or I7 (originally from Dr. Ted Hackstadt, NIH/NIAID Rocky Mountain Laboratories).They were used at 1:250 dilutions<br><br>Goat anti-Rabbit IgG (H+L) Cross-Adsorbed Secondary Antibody, Alexa Fluor™ 488 Invitrogen A11008 2420731 1:500

Goat anti-Mouse IgG (H+L) Cross-Adsorbed Secondary Antibody, Alexa Fluor™ 488 Invitrogen A11001 2513496 1:500

Goat anti-Rabbit IgG (H+L) Cross-Adsorbed Secondary Antibody, Alexa Fluor™ 647 Invitrogen A32733 XG349344 1:500

DAPI BD Biosciences 564907 1336109 1:2000

Alexa Fluor™ 568 Phalloidin Invitrogen A12380 2339924 1:500

Rickettsia japonica OMP Polyclonal Antibody Invitrogen PA5-117668 ZF4360061A 1:250

Mouse anti-Ubiquitin (FK1) Novus NBP3-11039 MU188322 1:250

Validation	The antibodies were used alongside negative controls, for example uninfected cells (as a negative control for Rickettsia). Similar validation was previously performed for the 14-13, I7205, and FK1 antibodies (Engström et al., 2019; Engström et al., 2021).

Eukaryotic cell lines

Policy information about cell lines and Sex and Gender in Research

Cell line source(s)	Vero cells were obtained from the UC Berkeley Cell Culture Facility. HMEC-1 cells were originally obtained from Dr. Matt Welch (UC Berkeley). BMDMs were derived from male or female mice.
Authentication	Vero and HMEC cells were authenticated at the UC Berkeley mass spectrometry facility.
Mycoplasma contamination	Mycoplasma was checked at least every month using a Mycoplasma Detection Kit (ABCAM Ab289834).
Commonly misidentified lines (See ICLAC register)	na

Animals and other research organisms

Policy information about studies involving animals; ARRIVE guidelines recommended for reporting animal research, and Sex and Gender in Research

Laboratory animals	Macrophages were derived from both male and female C57BL/6J mice.
Wild animals	n/a
Reporting on sex	BMDMs were derived from both male and female mice, and data from combined experiments include both male and female mice.
Field-collected samples	n/a
Ethics oversight	Animal research using mice was conducted under a protocol approved by the UC Irvine Institutional Animal Care and Use Committee (IACUC) in compliance with the Animal Welfare Act and other federal statutes relating to animals and experiments using animals (Burke lab animal use protocol AUP-22-005 and AUP-25-004). The UC Irvine IACUC is fully accredited by the Association for the Assessment and Accreditation of Laboratory Animal Care International and adheres to the principles of the Guide for the Care and use of Laboratory Animals.

Note that full information on the approval of the study protocol must also be provided in the manuscript.

Plants

Seed stocks	na
Novel plant genotypes	na
Authentication	na
